# Uncovering Mediating Mechanisms Linking Immune Cells to Systemic Lupus Erythematosus: Insights into Mendelian Randomization

**DOI:** 10.2174/0118715303407042251210161902

**Published:** 2026-04-03

**Authors:** Zenghui Liu, Lu Kuang, Jiaxing Zhao, Huayu Yin, Xuehui Liu, Dabin Liu, Shaoguo Wu, Limei Wu

**Affiliations:** 1 Department of Laboratory Medicine, The Affiliated Guangzhou Twelfth People's Hospital, Guangzhou Medical University, Guangzhou, Guangdong, China;; 2 Department of Immunology, Mudanjiang Medical University, Mudanjiang, Heilongjiang, China

**Keywords:** Immune cells, inflammatory proteins, plasma lipidome, plasma metabolites, systemic lupus erythematosus, Mendelian randomization

## Abstract

**Introduction:**

Systemic lupus erythematosus (SLE) involves dysregulated immune cell function, though its exact pathogenic mechanisms remain unclear.

**Methods:**

We performed a two-sample Mendelian randomization (MR) study using extensive GWAS summary statistics, covering 731 immune cell phenotypes, 91 inflammatory proteins, 179 lipid types, 1,400 blood metabolites, and SLE cases. The analysis aimed to evaluate causal associations between these variables and SLE. Additionally, we investigated possible mediating roles of inflammatory proteins, blood lipids, and metabolites through mediation analysis, with various sensitivity tests confirming the reliability of our findings.

**Results:**

We identified 20 immune cell phenotypes, 3 inflammatory proteins, 3 lipid types, and 17 blood metabolites with significant causal associations with SLE. Mediation analysis revealed that the protective effect of CCR2 on CD62L+ myeloid DCs against SLE was partly mediated by phosphatidylcholine (O−18:1_20:4) (12%) and sterol ester (27:1/20:3) (10.4%). Moreover, sphingomyelin (d18:1/20:0, d16:1/22:0) accounted for 14.7% of the protective effect of CD28 on CD45RA^−^ CD4^+^ cells, not Treg, against SLE.

**Discussion:**

These findings enhance our understanding of disease pathogenesis, indicating that modulating key metabolic pathways and immune regulatory nodes could represent promising therapeutic approaches. However, additional experimental studies are needed to validate these potential causal relationships.

**Conclusion:**

Using causal inference approaches, our study identifies immune cell-mediated mechanisms underlying SLE pathogenesis, involving inflammatory proteins, lipids, and metabolites. These findings suggest potential intervention pathways for further mechanistic exploration and therapeutic development.

## 1 INTRODUCTION

Systemic lupus erythematosus (SLE) is a complex autoimmune disorder characterized by immune dysregulation, pathogenic autoantibody production, and multi-organ damage [1, 2]. The development of SLE involves interactions between genetic susceptibility, environmental factors, and immune system abnormalities [1, 3, 4]. Recent studies have highlighted the roles of immune cell populations, inflammatory mediators, and metabolic changes in SLE pathogenesis, suggesting potential therapeutic targets [5, 6].

A key feature of SLE is significant dysregulation of immune cells [7, 8]. T cell dysfunction leads to overproduction of inflammatory cytokines, such as IL-17 and IFN-α, while impaired B cell regulation results in uncontrolled autoantibody production [9]. Additionally, myeloid cells (monocytes, dendritic cells, and macrophages) often exhibit pro-inflammatory activation, exacerbating tissue damage. Contemporary metabolic profiling studies have uncovered substantial modifications in lipid and metabolite signatures among SLE patients [10-12]. Metabolic studies have revealed substantial alterations in lipid and metabolite profiles in SLE patients, including reduced anti-inflammatory lipids and increased triacylglycerol levels, which may correlate with disease severity [13-15]. Immune-metabolic interactions are further demonstrated by cytokine-mediated metabolic changes; for example, IL-4 enhances lipid synthesis and storage in adipocytes while reducing circulating free fatty acids, influencing plasma triglyceride levels [16, 17]. Metabolomic analyses have also identified dysregulation in lipid, amino acid, and nucleotide metabolism in SLE patients, which may be associated with immune dysfunction and disease activity [18, 19]. These metabolic alterations could serve as potential biomarkers for SLE diagnosis and treatment [20, 21]. However, the exact mechanisms linking immune dysfunction and metabolic changes in SLE remain unclear.

Traditional observational studies are limited by confounding factors and reverse causation [22, 23]. Mendelian randomization (MR) analysis addresses these limitations by using genetic variants as instrumental variables, providing more robust causal inferences [24-26]. In this study, we employed a comprehensive MR approach to investigate causal relationships between immune cells, inflammatory proteins, metabolic profiles, and SLE risk. Mediation analyses were conducted to identify potential intermediate pathways, and reverse MR analyses were performed to confirm causal directions, enhancing the validity of our findings.

## 2 MATERIALS AND METHODS

### 2.1 Study Design and Type of Study

This study employed a two-sample Mendelian randomization design, a genetic epidemiological approach that uses instrumental variable analysis to infer causal relationships (Fig. **1**). The analysis utilized publicly available genome-wide association study (GWAS) summary statistics from European-ancestry populations. This study adheres to the Strengthening the Reporting of Observational Studies in Epidemiology (STROBE) guidelines for Mendelian Randomization (STROBE-MR) [27].

### 2.2 Data Sources

Summary statistics for SLE were obtained from the FinnGen R12 dataset (November 2024 release), comprising 1,306 SLE cases and 37,273 control samples [28]. Immune cell data were obtained from Orrù *et al*. (n = 3,757) [29], with dataset IDs ranging from GCST90001391 to GCST90002121. Inflammatory protein data (Zhao *et al*., n = 14,824) included 91 proteins analyzed *via* pQTL (IDs: GCST90274758 to GCST90274848) [30]. Plasma lipidome data (Ottensmann *et al*., n = 7,174) encompassed glycerides (GL, 44 species), glycerophospholipids (GP, 104 species), sphingolipids (SL, 15 species), and sterols (ST, 16 species), with accession identifiers spanning GCST90277238 to GCST90277416 [31]. Plasma metabolite data (Chen *et al*., n = 8,299) included 1,091 metabolites and 309 metabolite ratios measured *via* UPLC-MS/MS (Metabolon HD4), with dataset IDs GCST90199621 to GCST90201020 [32].

All GWAS data used in this study were obtained from publicly available summary-level datasets [33]. The original studies providing these data had received ethical approval from relevant institutional review boards, and all participants had given informed consent [34]. Since our analysis involved only secondary use of existing datasets, additional ethical approval was not required [35]. To avoid potential bias from sample overlap, we ensured that the SLE case-control cohorts were entirely independent from the datasets used for immune cells, inflammatory proteins, lipidomics, and metabolomics. This approach strengthened the reliability of our causal inferences.

### 2.3 SNP Selection and Instrumental Variable Validation

We selected single-nucleotide polymorphisms (SNPs) significantly associated with immune cells, inflammatory proteins, plasma lipids, metabolites, and SLE status (*P* < 5×10^8^) as instrumental variables (IVs) [36]. To ensure independence between IVs, we applied stringent linkage disequilibrium (LD) clumping (r^2^ < 0.001 within a 10,000 kb window) [37]. Variants with minor allele frequency (MAF) < 0.01 were excluded to avoid population stratification bias [38]. IV strength was evaluated using the F-statistic, and SNPs with F < 10 were removed to prevent weak instrument bias [39]. Outcome-associated SNPs were extracted from the IEU OpenGWAS and FinnGen databases [40]. Exposure and outcome datasets were harmonized, and ambiguous palindromic SNPs (A/T or G/C) were excluded [41]. Potential pleiotropic variants were identified using LDtrait (https://ldlink.nih.gov) and subsequently removed from the analysis [42].

For each SNP, we extracted the following information: chromosomal position, effect allele (EA), other allele (OA), effect allele frequency (EAF), effect size (β), standard error (SE), and association *P*-value [43]. The proportion of variance explained (R^2^) was calculated as: R^2^ = 2×EAF× (1-EAF) ×β^2^ / (2×EAF×(1-EAF) ×β^2^ + 2×EAF×(1-EAF) ×N×SE^2^), where N represents the effective sample size. Instrument strength was assessed using: F = R^2^×(N-2)/(1-R^2^) [44].

### 2.4 MR Analysis

All analyses were performed in R (v4.4.3) with the MR- PRESSO (v1.0) and TwoSampleMR (v0.6.8) packages. Causal estimation methods were selected based on SNP availability. When only one SNP was available, we employed the Wald ratio method for estimation; whereas in cases where two or more SNPs were available, the inverse variance weighted (IVW) method was used as the primary approach to assess the causal relationship between exposure and outcome [45]. Complementary MR approaches were implemented for sensitivity analyses, including MR-Egger regression, simple mode, weighted median, and weighted mode [46]. The Steiger directionality test was used to assess whether the hypothesized causal direction was consistent with the data. Quality control measures included: heterogeneity assessment (Cochran's Q test, *P*<0.05 threshold), pleiotropy evaluation (MR-PRESSO global test and MR-Egger intercept, *P*>0.05 indicating no bias), and leave-one-out analysis for identification of influential SNPs [47]. We implemented multiple testing corrections using two complementary approaches. First, multiple testing correction employed Bonferroni-adjusted thresholds by setting the following adjusted *P*-value thresholds: 6.84×10^−5^ (0.05/731), 3.57×10^−5^ (0.05/1400), 2.79×10^−4^ (0.05/179), and 5.49×10^−4^ (0.05/91) [48]. Associations meeting *P*<0.05 but not surpassing corrected thresholds were classified as suggestive. Second, to account for potential inflation of type I error rates due to multiple testing, we adjusted the primary IVW estimates using the Benjamini-Hochberg false discovery rate (FDR) correction. Associations with FDR-adjusted *P*-values < 0.1 were considered statistically significant, while those with nominal *P*-values < 0.05 but FDR ≥ 0.1 were regarded as suggestive evidence [49].

### 2.5 Mediation Analysis

Selected immune cells, plasma lipids, metabolites, and inflammatory proteins were identified based on significant causal associations with SLE (Bonferroni threshold *P* < 0.05, FDR > 0.1), while ensuring the absence of heterogeneity and pleiotropy among these factors. Second, the effects of immune cells on candidate mediators were assessed using identical selection criteria. Third, a two-step MR analysis was conducted to examine the total effect of immune cells on SLE (βall), the effect of immune cells on potential mediators, including plasma lipidome, metabolites, and inflammatory proteins (β_1_), and the effect of these mediators on SLE (β_2_). For positive βall, β_1_ and β_2_ must share direction (both positive/negative) [50]. Conversely, for negative βall, β_1_ and β_2_ must have opposing signs. Subsequently, the mediation effect was calculated using the product of coefficients method (β_1_×β_2_), and the proportion mediated was estimated as the ratio of the mediation effect to the total effect: (β_1_×β_2_) / βall. The 95% confidence interval (CI) for the mediation effect was determined using the Delta method [51]. Additionally, the direct effect was obtained by subtracting the mediation effect from the total effect: (βall – β_1_×β_2_). Mediation effects with *P*<0.05 were considered statistically significant [52].

### 2.6 Enrichment Analysis

In our investigation, we first identified SNPs from genomic datasets that showed significant correlations with immune cell profiles, SLE susceptibility, and clinical phenotypes. These candidate SNPs were systematically annotated using g:Profiler (https://biit.cs.ut.ee/gprofiler/snpense), a versatile bioinformatics resource that enables SNP-to-gene mapping, functional enrichment assessment, and cross-species ortholog alignment [53]. To characterize the biological functions of identified genes, we performed functional enrichment analysis using an integrated bioinformatics platform (https://www.bioinformatics.com.cn/) along with R packages clusterProfiler and org.Hs.eg.db for Gene Ontology (GO) and KEGG pathway analysis (*P* < 0.05). Additionally, we constructed a protein-protein interaction (PPI) network using GeneMANIA to investigate functional relationships and potential molecular mechanisms among these genes.

## 3 RESULTS

### 3.1 Causal Effects of Immune Cells on Systemic Lupus Erythematosus

Using two-sample MR analysis, we identified 20 immune cell phenotypes with causal associations with SLE. Among these, eight phenotypes exhibited protective effects, while twelve were associated with increased risk. Protective associations were observed for B cell subsets expressing CD20, including: CD20 on IgD^+^ (OR = 0.737, 95% CI: 0.602 ~ 0.904), CD20 on naive-mature (OR = 0.742, 95% CI: 0.610 ~ 0.903), and CD20 on IgD^+^ CD24^−^ (OR = 0.750, 95% CI: 0.600 ~ 0.936). These findings suggest that higher CD20 expression in specific B-cell populations may reduce SLE risk, providing genetic support for the therapeutic mechanism of anti-CD20 monoclonal antibodies (*e.g.*, rituximab). Conversely, several immune phenotypes were positively associated with SLE susceptibility, including: Unsw Mem %lymphocyte (OR=1.617, 95% CI:1.126 ~ 2.322), CD4 on EM CD4+ (OR=1.505, 95% CI:1.182 ~ 1.918), and CD25 on IgD+ CD24+ (OR=1.295, 95% CI:1.056 ~ 1.589). These associations suggest a potential role in SLE pathogenesis, possibly through dysregulated B cell and T cell activation (Fig. **2A**). Furthermore, the causal relationships were consistent across four additional MR methods (Supplementary Fig. **S1**), and additional sensitivity analyses supported the reliability of the findings, with no evidence of significant heterogeneity or horizontal pleiotropy (Supplementary Tables **S1** and **S2**).

We additionally examined potential bidirectional causality, assessing whether SLE might influence the 20 immune cell phenotypes. Reverse MR analysis indicated that CD4 on EM CD4+ and HLA-DR on myeloid DC exhibited significant reverse causal relationships with SLE, whereas the remaining 18 phenotypes showed no significant effects (Fig. **2B**).

### 3.2 Causal Effects of Inflammatory Proteins on Systemic Lupus Erythematosus

Forward MR analysis identified three inflammatory proteins significantly associated with SLE, including two risk factors and one protective factor (Fig. **3A**). Using the IVW method, genetically predicted higher levels of C-C motif chemokine 25 (CCL-25) were associated with a reduced risk of SLE (OR = 0.849, 95% CI: 0.756-0.952), suggesting a potential protective effect. Conversely, elevated levels of C-C motif chemokine 19 (CCL-19) (OR = 1.312, 95% CI: 1.049 ~ 1.642) and osteoprotegerin (OPG) (OR = 1.555, 95% CI: 1.128 ~ 2.143) were associated with an increased SLE risk. These findings nominate CCL-19 and OPG as potential serum biomarkers for SLE diagnosis or subtype stratification. Sensitivity analyses validated these associations, revealing no substantial heterogeneity or horizontal pleiotropic effects (Supplementary Tables **S3** and **S4**).

To assess reverse causation, we performed a reverse MR analysis evaluating the effects of SLE on the three inflammatory proteins. No significant causal associations were observed (Fig. **3B**), suggesting that SLE does not substantially alter circulating levels of these proteins.

### 3.3 Causal Effects of Plasma Lipidome on Systemic Lupus Erythematosus

Forward MR analysis identified three lipid species as protective causal factors against SLE (Fig. **4A**). The primary IVW analysis demonstrated that elevated levels of Phosphatidylcholine (O−18:1_20:4) (OR = 0.773, 95% CI: 0.611 ~ 0.979), Sterol ester (27:1/20:3) (OR = 0.789, 95% CI: 0.623 ~ 0.998), and Sphingomyelin (d40:1) (OR = 0.809, 95% CI: 0.668 ~ 0.981) were associated with a decreased risk of SLE. Measuring baseline lipid levels, particularly in high-risk individuals with a family history of SLE, may help predict susceptibility and facilitate early risk assessment and monitoring. Sensitivity analyses confirmed the reliability of the results, with no detectable heterogeneity or pleiotropy (Supplementary Tables **S5** and **S6**).

To evaluate potential reverse causation, we performed reverse MR analysis to assess the effect of SLE on the three lipid species. The results revealed no significant causal impact of SLE on these lipid levels (Fig. **4B**).

### 3.4 Causal Effects of Plasma Metabolites on Systemic Lupus Erythematosus

IVW analysis revealed 17 plasma metabolites with causal associations to SLE, comprising 10 protective and 7 risk-associated metabolites. Notably, glucose to N−stearoyl−sphingosine (d18:1 to 18:0) ratio, X−11880 levels, and N−palmitoyl−sphingosine (d18:1 to 16:0) to N−stearoyl−sphingosine (d18:1 to 18:0) ratio exhibited significant protective effects, with ORs of 0.641 (95% CI: 0.449 ~ 0.915), 0.650 (95% CI: 0.433 ~ 0.977), and 0.662 (95% CI: 0.502 ~ 0.873), respectively. In contrast, N−stearoyl−sphingosine (d18:1/18:0) levels, stearoyl sphingomyelin (d18:1/18:0) levels, and glycosyl ceramide (d18:1/20:0, d16:1/22:0) levels were associated with increased SLE risk, with ORs of 1.547 (95% CI: 1.112 ~ 2.151), and 1.513 (95% CI: 1.157 ~ 1.978), and1.321 (95% CI: 1.061 ~ 1.645) respectively (Fig. **5A**). These findings highlight significant causal relationships between sphingolipid metabolites (including ceramide derivatives and sphingomyelins) and SLE susceptibility. Quantification of these metabolites could potentially facilitate the development of early screening tools for high-risk populations.

Four complementary MR methods corroborated the IVW results (Supplementary Fig. **S2**), with sensitivity analyses confirming robustness (Supplementary Tables **S7** and **S8**). Reverse MR analysis revealed that SLE significantly influenced only N-stearoyl-sphingosine (d18:1/18:0) levels, with no causal effects on other metabolites (Fig. **5B**).

### 3.5 Mediation Analysis

To enhance causal inference, variables demonstrating reverse causation were excluded, followed by a two-step MR analysis of the remaining immune phenotypes, inflammatory proteins, plasma lipids, and metabolites. Initial screening identified specific inflammatory proteins and plasma components as potential mediators (Fig. **6**). Sensitivity analyses supported the robustness of these findings, with no evidence of significant heterogeneity or pleiotropy (Supplementary Tables **S9** and **S10**).

Subsequent mediation analysis evaluated the role of immune cells in influencing these mediators and their subsequent effects on SLE risk (Table **1**). The results indicated that CCR2 on CD62L+ myeloid DC exerted a protective effect against SLE, partially mediated by phosphatidylcholine (O−18:1_20:4) (12%) and sterol ester (27:1/20:3) (10.4%). Additionally, CCL25 mediated the protective effect of CD33dim HLA DR+ CD11b- %CD33dim HLA DR+ on SLE, with a mediation proportion of 3.58%. Moreover, sphingomyelin (d18:1/20:0, d16:1/22:0) levels mediated the protective effect of CD28 on CD45RA^−^ CD4, but not on Treg cells, against SLE, with a mediation proportion of 14.7%.

In summary, these findings demonstrate that inflammatory proteins and plasma components play a significant mediating role in the association between immune cell phenotypes and SLE, providing novel mechanistic insights into SLE pathogenesis.

### 3.6 Results of Enrichment Analysis

After converting the SNPs significantly associated with immune cells and SLE into genes, enrichment analysis was performed, as detailed in Fig. (**7**) and Supplementary Table **S11**. The top five clusters included the immune response−regulating cell surface receptor signaling pathway, the external side of the plasma membrane, cell killing, the inflammatory response to an antigenic stimulus, and immune receptor activity (Fig. **7A**). KEGG analysis highlighted pathways, including systemic lupus erythematosus, leishmaniasis, antigen processing and presentation, and adherens junction (Fig. **7B**). Significant gene loci were mapped to chromosomes (Supplementary Fig. **S3**), and their interactions were visualized in a GeneMANIA-derived PPI network (Supplementary Fig. **S4**).

## 4 DISCUSSION

Using MR analysis, we identified significant causal associations between SLE and immune cell phenotypes, inflammatory proteins, and plasma components (lipids/metabolites). Mediation analysis further revealed their potential intermediary roles in SLE pathogenesis, offering novel mechanistic insights and therapeutic targets.

We identified a bidirectional interplay between immune regulation and SLE development, suggesting both protective and pathogenic contributions. Certain immune cell phenotypes exhibit protective effects by limiting excessive immune activation, while others increase SLE risk by promoting immune dysregulation and inflammatory responses [54, 55]. Notably, abnormal activation of B cell subsets is closely linked to SLE onset, as observed in our findings [56]. For example, CD20 expression on IgD+ B cells was significantly associated with a reduced risk of SLE, whereas CD4 expression on effector memory T cells (EM CD4+) was linked to an increased risk. The protective role of CD20 on B cells may be attributed to its regulation of B cell activation and differentiation, preventing excessive immune responses [57]. Reduced CD20 expression could inhibit B-cell hyperactivation, thereby decreasing autoantibody production and immune complex deposition [58]. Higher CD4 levels on EM CD4+ cells could enhance Th1/Th17 polarization, boosting IFN-γ and IL-17 production that worsens inflammatory damage [59]. These findings suggest novel immunotherapeutic targets for SLE. For instance, monoclonal antibodies targeting CD20, such as rituximab, have been used in the treatment of SLE, though their efficacy varies among individuals [60]. Our study suggests that CD20 expression on specific B cell subsets may be more protective, warranting further investigation into their therapeutic potential.

This study, using MR analysis, identified three inflammatory proteins significantly associated with SLE: CCL-25, CCL-19, and OPG. Previous studies have shown that CCL-25 expression is altered in the immune microenvironment of SLE patients and is linked to Treg function and immune tolerance [61]. As a member of the chemokine family, CCL-25 primarily regulates the migration and localization of T and B cells [62]. The protective role of CCL-25 may involve Treg recruitment and subsequent suppression of pathogenic Th1/Th17 activation [63]. In contrast, the role of CCL-19 in dendritic cell-mediated immune activation aligns with the excessive inflammatory responses observed in SLE, consistent with its positive association with SLE risk [64]. CCL-19 primarily facilitates the migration of T cells and dendritic cells during immune responses [65]. In SLE, excessive immune cell aggregation at inflammatory sites is common, and CCL-19 overexpression may disrupt immune cell homeostasis, leading to abnormal accumulation in inflamed tissues [66]. These findings provide a theoretical basis for developing inhibitors targeting these inflammatory proteins. For example, inhibiting CCL-19 activity could potentially alleviate inflammatory responses and reduce SLE severity.

Emerging evidence suggests a critical role for plasma lipidome and metabolites in SLE. MR analysis identified three lipid species with a protective causal relationship with SLE: phosphatidylcholine (O−18:1_20:4), cholesteryl ester (27:1/20:3), and sphingomyelin (d40:1). Phosphatidylcholine is a key membrane component regulating cell signaling and communication [67]. In SLE, immune dysregulation leads to excessive autoantibody production, immune complex deposition, and subsequent tissue inflammation and damage [68]. The structural integrity of cell membranes, supported by phosphatidylcholine, is essential for maintaining cellular function, protecting against external stimuli, and resisting autoimmune attacks [69]. Cholesteryl ester, a key lipid for storage and transport, serves as an energy reservoir and contributes to membrane structure [70]. In immune cells, cholesteryl ester metabolism regulates processes, such as cell proliferation, differentiation, and cytokine secretion [71]. Balanced cholesteryl ester metabolism helps maintain immune cell homeostasis, preventing excessive activation or dysfunction, which could otherwise drive uncontrolled autoimmune responses in SLE [72]. Metabolomic analyses further revealed the pro-inflammatory effects of sphinganine, which may activate TLR4 signaling or disrupt autophagy, thereby exacerbating tissue damage [73]. The ratio of glucose to N-stearoyl sphinganine may modulate energy metabolism and oxidative stress, suppressing excessive immune cell activation and exerting protective effects [74]. These findings provide new evidence supporting the metabolic reprogramming theory in SLE. Metabolites identified in this study could serve as novel biomarkers for SLE diagnosis and disease activity assessment [75]. Furthermore, dietary or pharmacological interventions targeting these metabolites may improve patient prognosis.

The protective effects of specific immune cell phenotypes on SLE were partially mediated by plasma lipids/metabolites. For instance, the protective effect of CCR2 on CD62L^+^ myeloid dendritic cells is linked to phosphatidylcholine (O−18:1_20:4) and cholesteryl ester (27:1/20:3). Similarly, the protective effect of CD28 on CD45RA^−^ CD4^+^ non-regulatory T cells is associated with the suppression of sphingomyelin (d18:1/20:0, d16:1/22:0 and the elevation of X-21467. These immune-metabolic interactions may offer therapeutic avenues for SLE. These mediation pathways could serve as potential therapeutic targets, where modulating them might enhance the protective effects of immune cells or mitigate their risk effects. To explore how immune cells influence SLE development, we analyzed SNPs linked to both immune cells and SLE. SNPs associated with both immune cells and SLE were significantly enriched in immune regulatory pathways (GO/KEGG). Protein-protein interaction network analysis identified hub genes at the intersection of immune function and SLE-associated pathways, highlighting biologically plausible candidates for further mechanistic investigation.

Although this study provides robust analyses, several limitations should be acknowledged. First, the GWAS summary data were obtained exclusively from European-ancestry cohorts. While this approach minimizes population stratification bias, it may limit the generalizability of the findings to other populations. Notably, systemic lupus erythematosus exhibits racial disparities in prevalence, clinical manifestations, and serological profiles. Future research should validate the key findings in independent multi-ethnic cohorts. Second, despite employing multiple sensitivity analyses to assess pleiotropy and heterogeneity, Mendelian randomization remains susceptible to residual confounding from unmeasured or unknown factors. Further experimental validation is necessary to confirm these causal relationships. Third, while the study identifies statistically significant mediating effects, the underlying biological mechanisms (*e.g.*, how specific metabolites mediate immune cell function) require further validation through experimental models.

The present study should be interpreted alongside our previous Mendelian randomization investigation of Systemic Lupus Erythematosus (SLE), which employed a similar analytical framework using publicly available GWAS datasets. Although both studies utilized standard Mendelian randomization methodologies and shared certain publicly available GWAS resources, they addressed distinct disease entities, investigated different biological questions, analyzed independent exposure–outcome datasets, and generated separate analyses, results, and scientific conclusions. The current study therefore represents an independent investigation rather than an extension or duplication of the previous work [76].

## CONCLUSION

This study employed multidimensional Mendelian randomization analysis to investigate causal relationships and mediation mechanisms involving immune cells, inflammatory proteins, lipidomic profiles, and metabolites in systemic lupus erythematosus. The findings provide new insights into disease pathogenesis, suggesting that targeting key metabolic pathways and immune regulatory nodes may offer potential therapeutic strategies. However, further experimental validation is required to confirm these causal associations.

## Figures and Tables

**Fig. (1):**
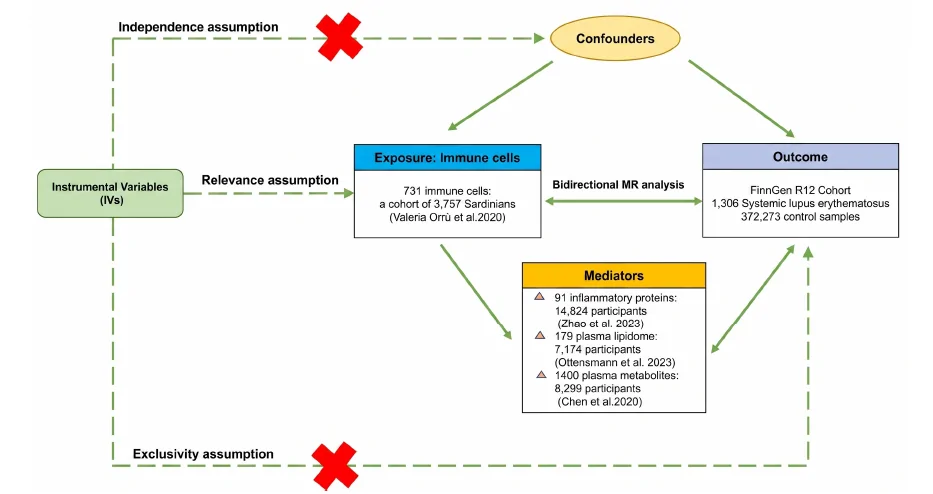
Study flowchart. Relevance: IVs show a significant exposure association. Independence: IVs must remain unconfounded by exposure-outcome covariates. Exclusivity: IVs influence outcome exclusively through exposure pathways.

**Fig. (2):**
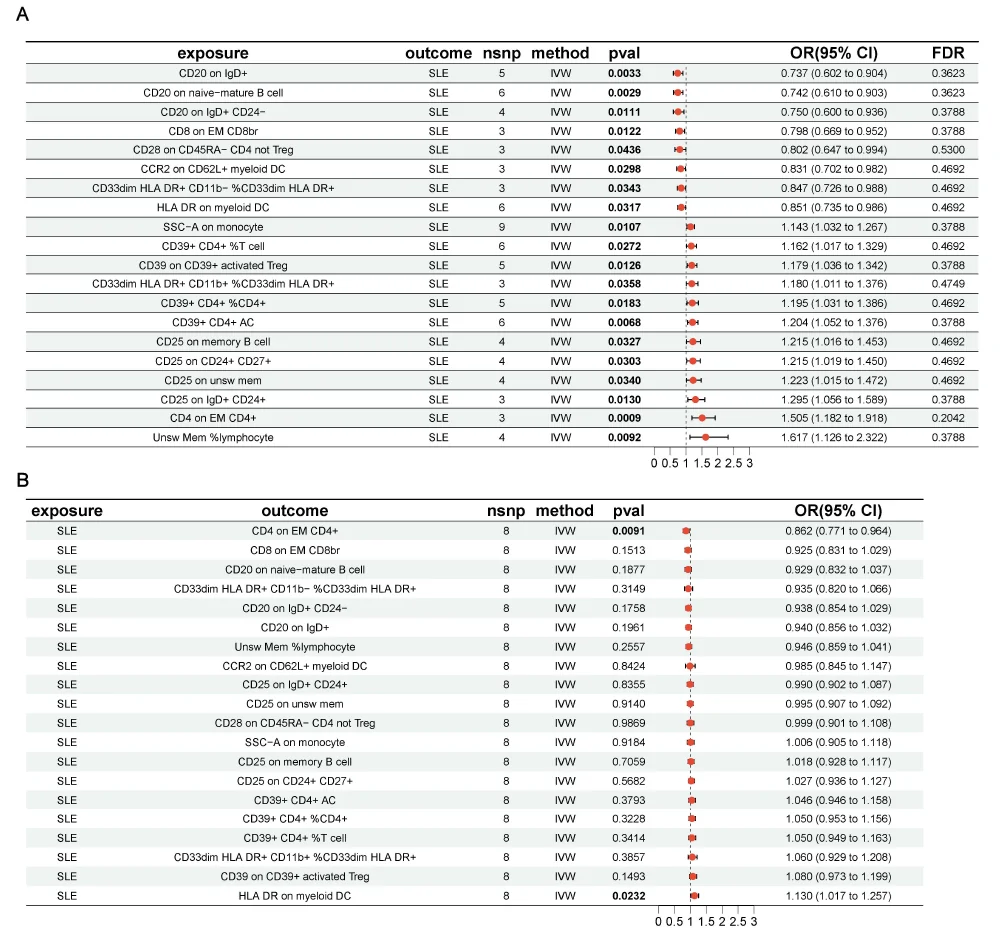
Mendelian randomization results presented as forest plots. (**A**) Causal effects of immune cells on SLE. (**B**) Reverse-direction analysis of SLE effects on immune cells. The odds ratios (ORs), 95% confidence intervals (CIs), and *P*-values were calculated for each Mendelian randomization analysis method. A *P*-value < 0.05 was considered statistically significant. **Abbreviations:** nsnp: number of single-nucleotide polymorphisms; IVW: inverse-variance weighted. SSC-A: side scatter-area, a flow cytometry parameter indicating cell internal complexity.

**Fig. (3):**
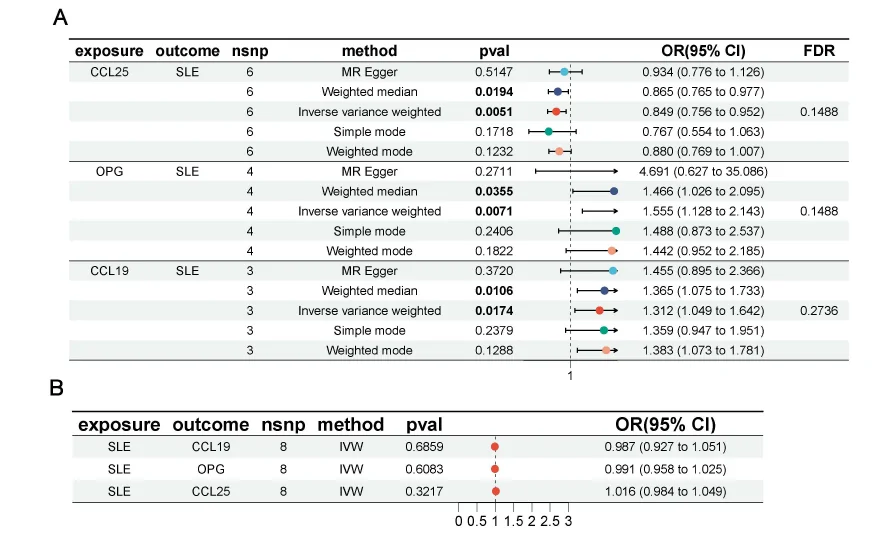
Mendelian randomization results presented as forest plots. (**A**) Causal effects of inflammatory proteins on SLE. (**B**) Reverse-direction analysis of SLE effects on inflammatory proteins. The odds ratios (ORs), 95% confidence intervals (CIs), and *P*-values were calculated for each Mendelian randomization analysis method. A *P*-value < 0.05 was considered statistically significant. **Abbreviations:** nsnp: number of single-nucleotide polymorphisms; IVW: inverse-variance weighted.

**Fig. (4):**
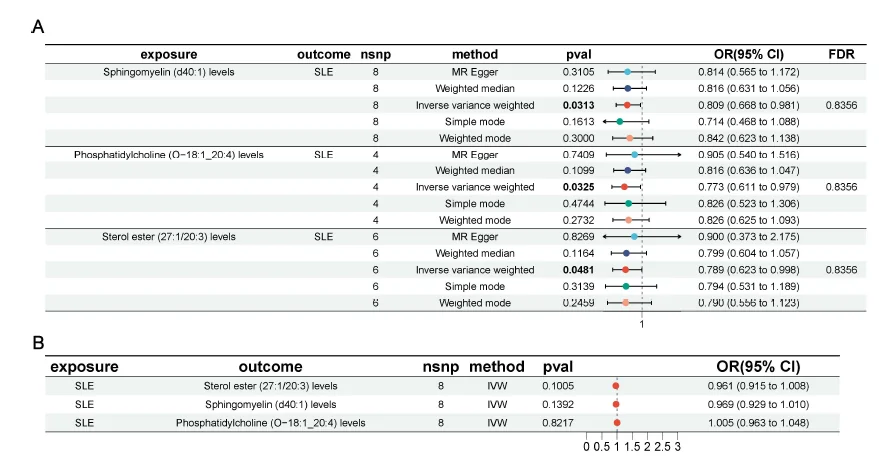
Mendelian randomization results presented as forest plots. (**A**) Causal effects of plasma lipidome on SLE. (**B**) Reverse-direction analysis of SLE effects on plasma lipidome. The odds ratios (ORs), 95% confidence intervals (CIs), and *P*-values were calculated for each Mendelian randomization analysis method. A *p*-value < 0.05 was considered statistically significant. **Abbreviations:** nsnp: number of single-nucleotide polymorphisms; IVW: inverse-variance weighted.

**Fig. (5):**
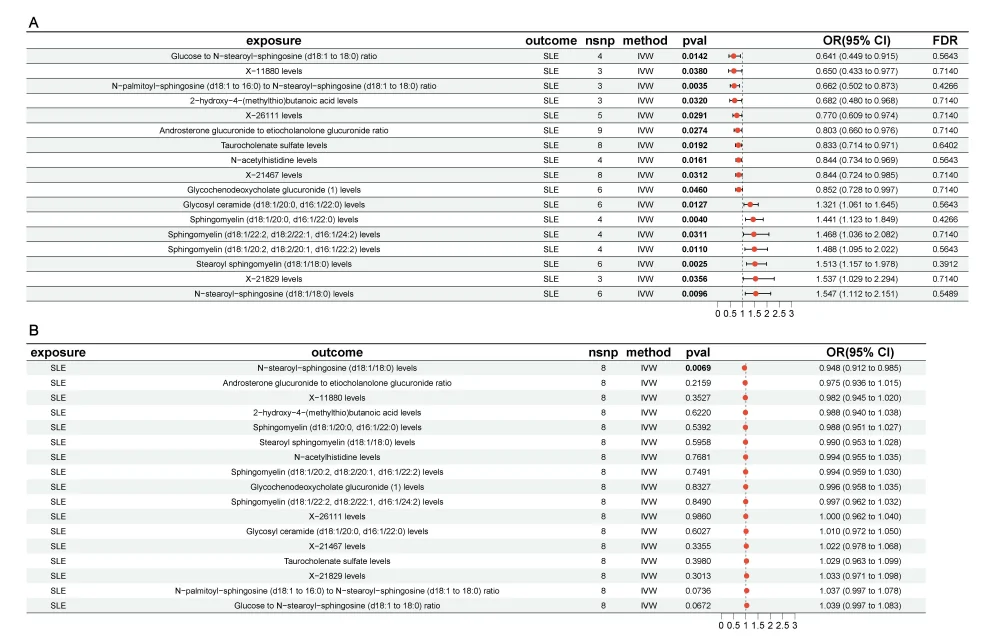
Mendelian randomization results presented as forest plots. (**A**) Causal effects of plasma metabolites on SLE. (**B**) Reverse-direction analysis of SLE effects on plasma metabolites. The odds ratios (ORs), 95% confidence intervals (CIs), and *P*-values were calculated for each Mendelian randomization analysis method. A *P*-value < 0.05 was considered statistically significant. **Abbreviations:** nsnp: number of single-nucleotide polymorphisms; IVW: inverse-variance weighted.

**Fig. (6):**
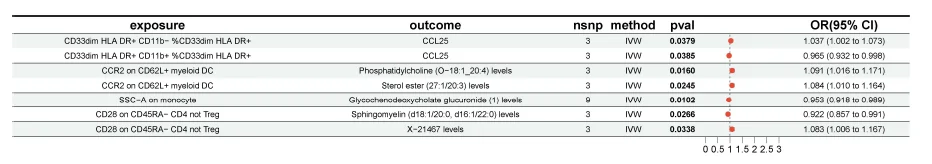
The results of the mediation analysis. The forest plot demonstrates the mediation patterns of “immune cells- plasma lipids /inflammatory proteins/ metabolites-SLE” in the two-step Mendelian randomization analysis. The odds ratios (ORs), 95% confidence intervals (CIs), and *P*-values were calculated for each Mendelian randomization analysis method. A *P*-value < 0.05 was considered statistically significant. **Abbreviations:** nsnp: number of single-nucleotide polymorphisms; IVW: inverse-variance weighted. SSC-A: side scatter-area, a flow cytometry parameter indicating cell internal complexity.

**Fig. (7):**
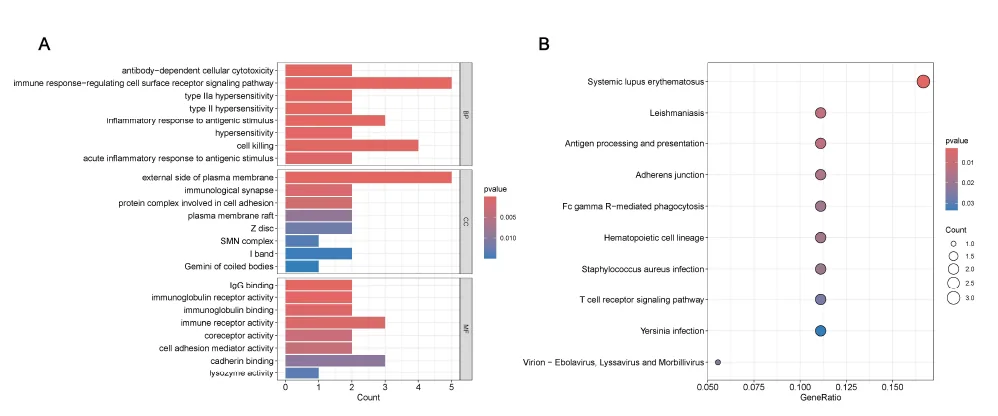
Enrichment analysis. (**A**) GO analysis, including Biological Processes (BPs), Molecular Functions (MFs), and Cellular Components (CCs). (**B**) KEGG pathway enrichment analysis.

**Table 1:** Mediation effects with proportion estimates for each candidate mediator.

**Immune Cell**	**Mediator**	**Outcome**	**Mediated Effect**	**Mediated Proportion**	**Direct Effect**	***P*-value**
CD33dim HLA DR+ CD11b- %CD33dim HLA DR+	CCL25	SLE	-0.00595 (-0.0117, -0.000197)	3.58% (7.04%, 0.119%)	-0.160	0.043
CD33dim HLA DR+ CD11b+ %CD33dim HLA DR+	CCL25	SLE	0.00592 (0.000179, 0.0117)	3.58% (0.108%, 7.06%)	0.159	0.043
CCR2 on CD62L+ myeloid DC	Phosphatidylcholine (O-18:1_20:4)	SLE	-0.0223 (-0.0415, -0.00316)	12% (22.4%, 1.7%)	-0.163	0.022
CCR2 on CD62L+ myeloid DC	Sterol ester (27:1/20:3)	SLE	-0.0192 (-0.037, -0.00152)	10.4% (19.9%, 0.818%)	-0.166	0.033
SSC-A on monocyte	Glycochenodeoxycholate glucuronide (1)	SLE	0.00774 (0.00157, 0.0139)	5.78% (1.18%, 10.4%)	0.126	0.014
CD28 on CD45RA- CD4 not Treg	Sphingomyelin (d18:1/20:2, d18:2/20:1, d16:1/22:2)	SLE	-0.0325 (-0.0618, -0.00317)	14.7% (27.9%, 1.43%)	-0.189	0.030
CD28 on CD45RA- CD4 not Treg	X-21467	SLE	-0.0136 (-0.0274, 0.000297)	6.13% (12.4%, -0.134%)	-0.208	0.055

**Abbreviations:** SSC-A: side scatter-area, a flow cytometry parameter indicating cell internal complexity.

## Data Availability

The data and supportive information are available within the article.

## LIST OF ABBREVIATIONS

SLE: Systemic Lupus Erythematosus
MR: Mendelian Randomization
IVW: Inverse Variance Weighted
IVs: Instrumental Variables
SNPs: Single-Nucleotide Polymorphisms
LD: Linkage Disequilibrium
CCL-25: C-C Motif Chemokine 25
OPG: Osteoprotegerin
CCL-19: C-C Motif Chemokine 19
PPI: Protein–Protein Interaction
GWAS: Genome-Wide Association Studies
DCs: Dendritic Cells
GL: Glycerides
GP: Glycerophospholipids
SL: Sphingolipids
ST: Sterols
EA: Effect Allele
OA: Other Allele
EAF: Effect Allele Frequency
β: Effect Sizes
SE: Standard Error
SSC-A: Side Scatter-Area
OR: Odds Ratio
CI: Confidence Interval

## STANDARDS OF REPORTING

STROBE-MR guidelines were followed.
