# Supplementary material for: Uncovering Mediating Mechanisms Linking Immune Cells to Systemic Lupus Erythematosus: Insights into Mendelian Randomization
Source: Endocr Metab Immune Disord Drug Targets. 2026 Apr 3;26:E18715303407042. doi: 10.2174/0118715303407042251210161902 (PMC13662731; doi:10.2174/0118715303407042251210161902)
Supplement: Supplementary file 1 [file EMIDDT-26-E18715303407042_SD1.pdf]

Supplementary Material

Uncovering Mediating Mechanisms Linking Immune Cells to Systemic Lupus Erythematosus: Insights into Mendelian Randomization

Zenghui Liu[1], Lu Kuang[2], Jiaxing Zhao[1], Huayu Yin[1], Xuehui Liu[2], Dabin Liu[2], Shaoguo Wu[2,*,#] and Limei WU[1,2,*,#]

[1]Department of Immunology, Mudanjiang Medical University, Mudanjiang, Heilongjiang, China; [2]Department of Laboratory Medicine, The Affiliated Guangzhou Twelfth People's Hospital, Guangzhou Medical University, Guangzhou, Guangdong, China

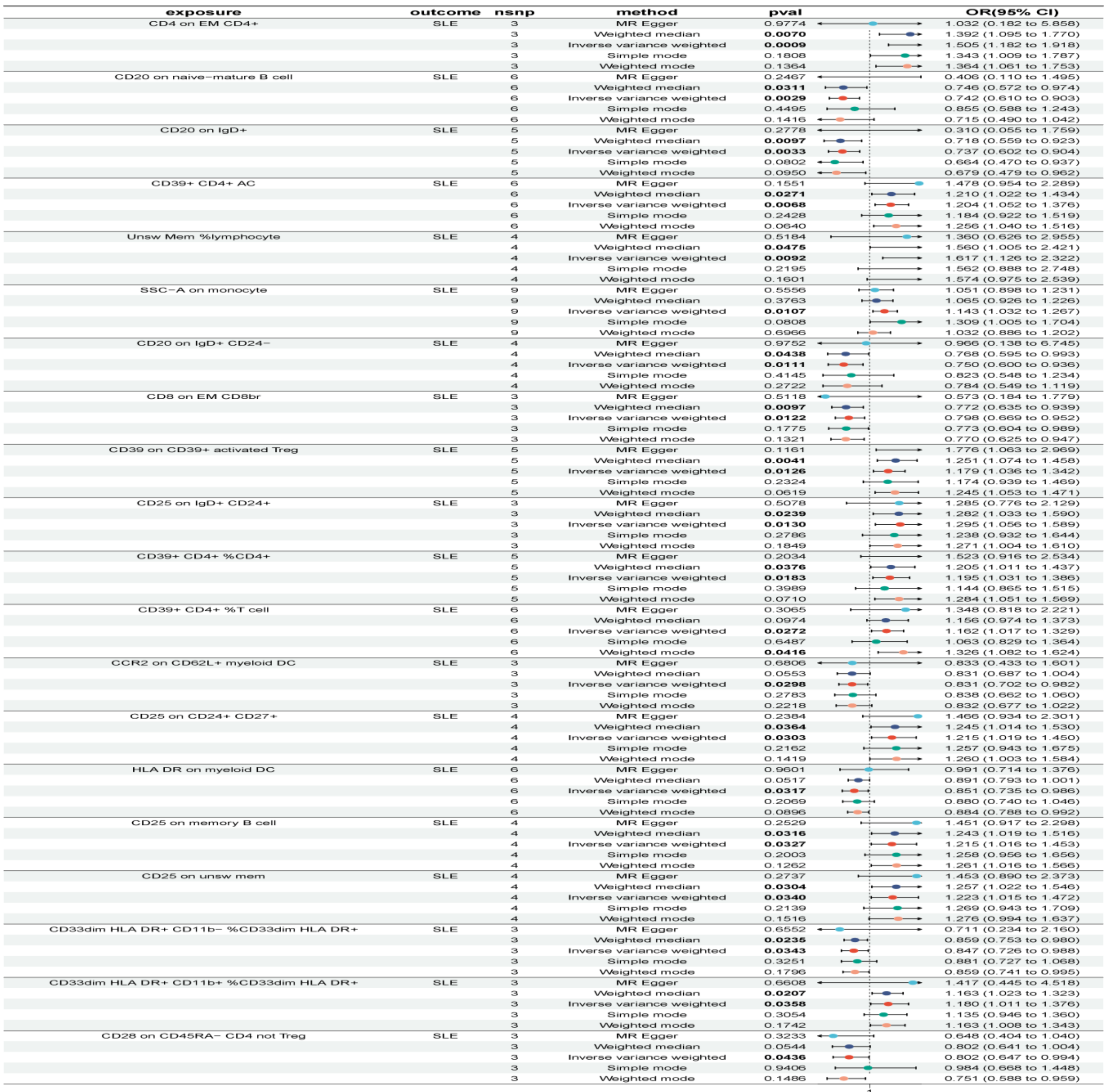

exposure	outcome	nsnp	method	pval	OR(95% CI)
CD4 on EM CD4+	SLE	3	MR Egger	0.9774	1.032 (0.182 to 5.858)
		3	Weighted median	**0.0070**	1.392 (1.095 to 1.770)
		3	Inverse variance weighted	**0.0009**	1.505 (1.182 to 1.918)
		3	Simple mode	0.1808	1.343 (1.009 to 1.787)
		3	Weighted mode	0.1364	1.364 (1.061 to 1.753)
CD20 on naive−mature B cell	SLE	6	MR Egger	0.2467	0.406 (0.110 to 1.495)
		6	Weighted median	**0.0311**	0.746 (0.572 to 0.974)
		6	Inverse variance weighted	**0.0029**	0.742 (0.610 to 0.903)
		6	Simple mode	0.4495	0.855 (0.588 to 1.243)
		6	Weighted mode	0.1416	0.715 (0.490 to 1.042)
CD20 on IgD+	SLE	5	MR Egger	0.2778	0.310 (0.055 to 1.759)
		5	Weighted median	**0.0097**	0.718 (0.559 to 0.923)
		5	Inverse variance weighted	**0.0033**	0.737 (0.602 to 0.904)
		5	Simple mode	0.0802	0.664 (0.470 to 0.937)
		5	Weighted mode	0.0950	0.679 (0.479 to 0.962)
CD39+ CD4+ AC	SLE	6	MR Egger	0.1551	1.478 (0.954 to 2.289)
		6	Weighted median	**0.0271**	1.210 (1.022 to 1.434)
		6	Inverse variance weighted	**0.0068**	1.204 (1.052 to 1.376)
		6	Simple mode	0.2428	1.184 (0.922 to 1.519)
		6	Weighted mode	0.0640	1.256 (1.040 to 1.516)
Unsw Mem %lymphocyte	SLE	4	MR Egger	0.5184	1.360 (0.626 to 2.955)
		4	Weighted median	**0.0475**	1.560 (1.005 to 2.421)
		4	Inverse variance weighted	**0.0092**	1.617 (1.126 to 2.322)
		4	Simple mode	0.2195	1.562 (0.888 to 2.748)
		4	Weighted mode	0.1601	1.574 (0.975 to 2.539)
SSC−A on monocyte	SLE	9	MR Egger	0.5556	1.051 (0.898 to 1.231)
		9	Weighted median	0.3763	1.065 (0.926 to 1.226)
		9	Inverse variance weighted	**0.0107**	1.143 (1.032 to 1.267)
		9	Simple mode	0.0808	1.309 (1.005 to 1.704)
		9	Weighted mode	0.6966	1.032 (0.886 to 1.202)
CD20 on IgD+ CD24−	SLE	4	MR Egger	0.9752	0.966 (0.138 to 6.745)
		4	Weighted median	**0.0438**	0.768 (0.595 to 0.993)
		4	Inverse variance weighted	**0.0111**	0.750 (0.600 to 0.936)
		4	Simple mode	0.4145	0.823 (0.548 to 1.234)
		4	Weighted mode	0.2722	0.784 (0.549 to 1.119)
CD8 on EM CD8br	SLE	3	MR Egger	0.5118	0.573 (0.184 to 1.779)
		3	Weighted median	**0.0097**	0.772 (0.635 to 0.939)
		3	Inverse variance weighted	**0.0122**	0.798 (0.669 to 0.952)
		3	Simple mode	0.1775	0.773 (0.604 to 0.989)
		3	Weighted mode	0.1321	0.770 (0.625 to 0.947)
CD39 on CD39+ activated Treg	SLE	5	MR Egger	0.1161	1.776 (1.063 to 2.969)
		5	Weighted median	**0.0041**	1.251 (1.074 to 1.458)
		5	Inverse variance weighted	**0.0126**	1.179 (1.036 to 1.342)
		5	Simple mode	0.2324	1.174 (0.939 to 1.469)
		5	Weighted mode	0.0619	1.245 (1.053 to 1.471)
CD25 on IgD+ CD24+	SLE	3	MR Egger	0.5078	1.285 (0.776 to 2.129)
		3	Weighted median	**0.0239**	1.282 (1.033 to 1.590)
		3	Inverse variance weighted	**0.0130**	1.295 (1.056 to 1.589)
		3	Simple mode	0.2786	1.238 (0.932 to 1.644)
		3	Weighted mode	0.1849	1.271 (1.004 to 1.610)
CD39+ CD4+ %CD4+	SLE	5	MR Egger	0.2034	1.523 (0.916 to 2.534)
		5	Weighted median	**0.0376**	1.205 (1.011 to 1.437)
		5	Inverse variance weighted	**0.0183**	1.195 (1.031 to 1.386)
		5	Simple mode	0.3989	1.144 (0.865 to 1.515)
		5	Weighted mode	0.0710	1.284 (1.051 to 1.569)
CD39+ CD4+ %T cell	SLE	6	MR Egger	0.3065	1.348 (0.818 to 2.221)
		6	Weighted median	0.0974	1.156 (0.974 to 1.373)
		6	Inverse variance weighted	**0.0272**	1.162 (1.017 to 1.329)
		6	Simple mode	0.6487	1.063 (0.829 to 1.364)
		6	Weighted mode	**0.0416**	1.326 (1.082 to 1.624)
CCR2 on CD62L+ myeloid DC	SLE	3	MR Egger	0.6806	0.833 (0.433 to 1.601)
		3	Weighted median	0.0553	0.831 (0.687 to 1.004)
		3	Inverse variance weighted	**0.0298**	0.831 (0.702 to 0.982)
		3	Simple mode	0.2783	0.838 (0.662 to 1.060)
		3	Weighted mode	0.2218	0.832 (0.677 to 1.022)
CD25 on CD24+ CD27+	SLE	4	MR Egger	0.2384	1.466 (0.934 to 2.301)
		4	Weighted median	**0.0364**	1.245 (1.014 to 1.530)
		4	Inverse variance weighted	**0.0303**	1.215 (1.019 to 1.450)
		4	Simple mode	0.2162	1.257 (0.943 to 1.675)
		4	Weighted mode	0.1419	1.260 (1.003 to 1.584)
HLA DR on myeloid DC	SLE	6	MR Egger	0.9601	0.991 (0.714 to 1.376)
		6	Weighted median	0.0517	0.891 (0.793 to 1.001)
		6	Inverse variance weighted	**0.0317**	0.851 (0.735 to 0.986)
		6	Simple mode	0.2069	0.880 (0.740 to 1.046)
		6	Weighted mode	0.0896	0.884 (0.788 to 0.992)
CD25 on memory B cell	SLE	4	MR Egger	0.2529	1.451 (0.917 to 2.298)
		4	Weighted median	**0.0316**	1.243 (1.019 to 1.516)
		4	Inverse variance weighted	**0.0327**	1.215 (1.016 to 1.453)
		4	Simple mode	0.2003	1.258 (0.956 to 1.656)
		4	Weighted mode	0.1262	1.261 (1.016 to 1.566)
CD25 on unsw mem	SLE	4	MR Egger	0.2737	1.453 (0.890 to 2.373)
		4	Weighted median	**0.0304**	1.257 (1.022 to 1.546)
		4	Inverse variance weighted	**0.0340**	1.223 (1.015 to 1.472)
		4	Simple mode	0.2139	1.269 (0.943 to 1.709)
		4	Weighted mode	0.1516	1.276 (0.994 to 1.637)
CD33dim HLA DR+ CD11b− %CD33dim HLA DR+	SLE	3	MR Egger	0.6552	0.711 (0.234 to 2.160)
		3	Weighted median	**0.0235**	0.859 (0.753 to 0.980)
		3	Inverse variance weighted	**0.0343**	0.847 (0.726 to 0.988)
		3	Simple mode	0.3251	0.881 (0.727 to 1.068)
		3	Weighted mode	0.1796	0.859 (0.741 to 0.995)
CD33dim HLA DR+ CD11b+ %CD33dim HLA DR+	SLE	3	MR Egger	0.6608	1.417 (0.445 to 4.518)
		3	Weighted median	**0.0207**	1.163 (1.023 to 1.323)
		3	Inverse variance weighted	**0.0358**	1.180 (1.011 to 1.376)
		3	Simple mode	0.3054	1.135 (0.946 to 1.360)
		3	Weighted mode	0.1742	1.163 (1.008 to 1.343)
CD28 on CD45RA− CD4 not Treg	SLE	3	MR Egger	0.3233	0.648 (0.404 to 1.040)
		3	Weighted median	0.0544	0.802 (0.641 to 1.004)
		3	Inverse variance weighted	**0.0436**	0.802 (0.647 to 0.994)
		3	Simple mode	0.9406	0.984 (0.668 to 1.448)
		3	Weighted mode	0.1486	0.751 (0.588 to 0.959)

Fig. (S1).

exposure	outcome	nsnp	method	pval	OR(95% CI)
Stearoyl sphingomyelin (d18:1/18:0) levels	SLE	6	MR Egger	0.1969	1.795 (0.855 to 3.767)
		6	Weighted median	**0.0073**	1.569 (1.129 to 2.181)
		6	Inverse variance weighted	**0.0025**	1.513 (1.157 to 1.978)
		6	Simple mode	0.1038	1.571 (1.006 to 2.454)
		6	Weighted mode	0.0554	1.588 (1.103 to 2.287)
N−palmitoyl−sphingosine (d18:1 to 16:0) to N−stearoyl−sphingosine (d18:1 to 18:0) ratio	SLE	3	MR Egger	0.6298	0.760 (0.335 to 1.722)
		3	Weighted median	**0.0097**	0.683 (0.512 to 0.912)
		3	Inverse variance weighted	**0.0035**	0.662 (0.502 to 0.873)
		3	Simple mode	0.2056	0.692 (0.468 to 1.022)
		3	Weighted mode	0.1447	0.685 (0.499 to 0.941)
Sphingomyelin (d18:1/20:0, d16:1/22:0) levels	SLE	4	MR Egger	0.6936	1.331 (0.388 to 4.569)
		4	Weighted median	**0.0106**	1.465 (1.093 to 1.965)
		4	Inverse variance weighted	**0.0040**	1.441 (1.123 to 1.849)
		4	Simple mode	0.6079	1.131 (0.741 to 1.726)
		4	Weighted mode	0.0996	1.494 (1.070 to 2.086)
N−stearoyl−sphingosine (d18:1/18:0) levels	SLE	6	MR Egger	0.1579	2.680 (0.880 to 8.162)
		6	Weighted median	**0.0117**	1.604 (1.111 to 2.316)
		6	Inverse variance weighted	**0.0096**	1.547 (1.112 to 2.151)
		6	Simple mode	0.1131	1.762 (0.988 to 3.141)
		6	Weighted mode	0.0652	1.659 (1.089 to 2.529)
Sphingomyelin (d18:1/20:2, d18:2/20:1, d16:1/22:2) levels	SLE	4	MR Egger	0.7843	1.170 (0.437 to 3.135)
		4	Weighted median	**0.0212**	1.495 (1.062 to 2.104)
		4	Inverse variance weighted	**0.0110**	1.488 (1.095 to 2.022)
		4	Simple mode	0.1249	1.744 (1.041 to 2.921)
		4	Weighted mode	0.2479	1.322 (0.902 to 1.939)
Glycosyl ceramide (d18:1/20:0, d16:1/22:0) levels	SLE	6	MR Egger	0.7700	1.114 (0.567 to 2.189)
		6	Weighted median	**0.0264**	1.360 (1.037 to 1.785)
		6	Inverse variance weighted	**0.0127**	1.321 (1.061 to 1.645)
		6	Simple mode	0.1202	1.586 (0.978 to 2.570)
		6	Weighted mode	0.4010	1.165 (0.841 to 1.613)
Glucose to N−stearoyl−sphingosine (d18:1 to 18:0) ratio	SLE	4	MR Egger	0.2568	0.263 (0.050 to 1.392)
		4	Weighted median	**0.0122**	0.573 (0.370 to 0.885)
		4	Inverse variance weighted	**0.0142**	0.641 (0.449 to 0.915)
		4	Simple mode	0.1439	0.539 (0.291 to 0.998)
		4	Weighted mode	0.1061	0.541 (0.319 to 0.915)
N−acetylhistidine levels	SLE	4	MR Egger	0.3886	0.857 (0.650 to 1.130)
		4	Weighted median	**0.0384**	0.852 (0.732 to 0.991)
		4	Inverse variance weighted	**0.0161**	0.844 (0.734 to 0.969)
		4	Simple mode	0.2271	0.849 (0.687 to 1.049)
		4	Weighted mode	0.1355	0.852 (0.729 to 0.995)
Taurocholenate sulfate levels	SLE	8	MR Egger	0.6782	0.929 (0.666 to 1.296)
		8	Weighted median	**0.0299**	0.819 (0.683 to 0.981)
		8	Inverse variance weighted	**0.0192**	0.833 (0.714 to 0.971)
		8	Simple mode	0.0920	0.728 (0.530 to 1.001)
		8	Weighted mode	0.0769	0.813 (0.668 to 0.989)
Androsterone glucuronide to etiocholanolone glucuronide ratio	SLE	9	MR Egger	0.8763	0.976 (0.730 to 1.305)
		9	Weighted median	0.1728	0.840 (0.655 to 1.079)
		9	Inverse variance weighted	**0.0274**	0.803 (0.660 to 0.976)
		9	Simple mode	0.6192	0.913 (0.645 to 1.291)
		9	Weighted mode	0.3346	0.870 (0.668 to 1.135)
X−26111 levels	SLE	5	MR Egger	0.9492	0.979 (0.532 to 1.801)
		5	Weighted median	0.0764	0.780 (0.593 to 1.027)
		5	Inverse variance weighted	**0.0291**	0.770 (0.609 to 0.974)
		5	Simple mode	0.1580	0.700 (0.468 to 1.048)
		5	Weighted mode	0.3144	0.847 (0.639 to 1.124)
Sphingomyelin (d18:1/22:2, d18:2/22:1, d16:1/24:2) levels	SLE	4	MR Egger	0.3889	1.641 (0.674 to 3.992)
		4	Weighted median	**0.0472**	1.518 (1.005 to 2.293)
		4	Inverse variance weighted	**0.0311**	1.468 (1.036 to 2.082)
		4	Simple mode	0.2125	1.555 (0.899 to 2.691)
		4	Weighted mode	0.1678	1.533 (0.965 to 2.434)
X−21467 levels	SLE	8	MR Egger	0.0600	0.695 (0.511 to 0.946)
		8	Weighted median	**0.0499**	0.835 (0.697 to 1.000)
		8	Inverse variance weighted	**0.0312**	0.844 (0.724 to 0.985)
		8	Simple mode	0.1062	0.757 (0.565 to 1.016)
		8	Weighted mode	0.0779	0.826 (0.689 to 0.990)
2−hydroxy−4−(methylthio)butanoic acid levels	SLE	3	MR Egger	0.8340	0.736 (0.077 to 7.023)
		3	Weighted median	0.0700	0.701 (0.477 to 1.029)
		3	Inverse variance weighted	**0.0320**	0.682 (0.480 to 0.968)
		3	Simple mode	0.2880	0.714 (0.450 to 1.132)
		3	Weighted mode	0.2988	0.705 (0.431 to 1.154)
X−21829 levels	SLE	3	MR Egger	0.7944	1.484 (0.147 to 14.980)
		3	Weighted median	0.2096	1.360 (0.841 to 2.199)
		3	Inverse variance weighted	**0.0356**	1.537 (1.029 to 2.294)
		3	Simple mode	0.5475	1.262 (0.668 to 2.382)
		3	Weighted mode	0.5593	1.256 (0.660 to 2.388)
X−11880 levels	SLE	3	MR Egger	0.9899	0.984 (0.141 to 6.886)
		3	Weighted median	0.0960	0.666 (0.412 to 1.075)
		3	Inverse variance weighted	**0.0380**	0.650 (0.433 to 0.977)
		3	Simple mode	0.3102	0.668 (0.372 to 1.201)
		3	Weighted mode	0.3056	0.681 (0.392 to 1.183)
Glycochenodeoxycholate glucuronide (1) levels	SLE	6	MR Egger	0.1985	0.759 (0.535 to 1.078)
		6	Weighted median	0.0942	0.856 (0.713 to 1.027)
		6	Inverse variance weighted	**0.0460**	0.852 (0.728 to 0.997)
		6	Simple mode	0.3013	0.873 (0.693 to 1.100)
		6	Weighted mode	0.1466	0.862 (0.728 to 1.021)

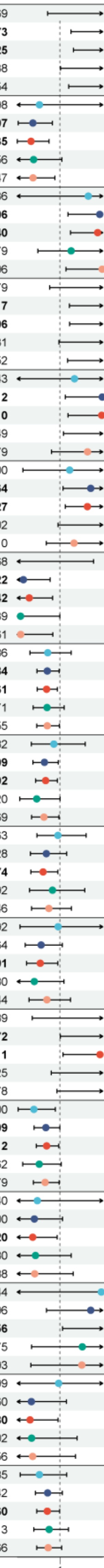

Fig. (S2).

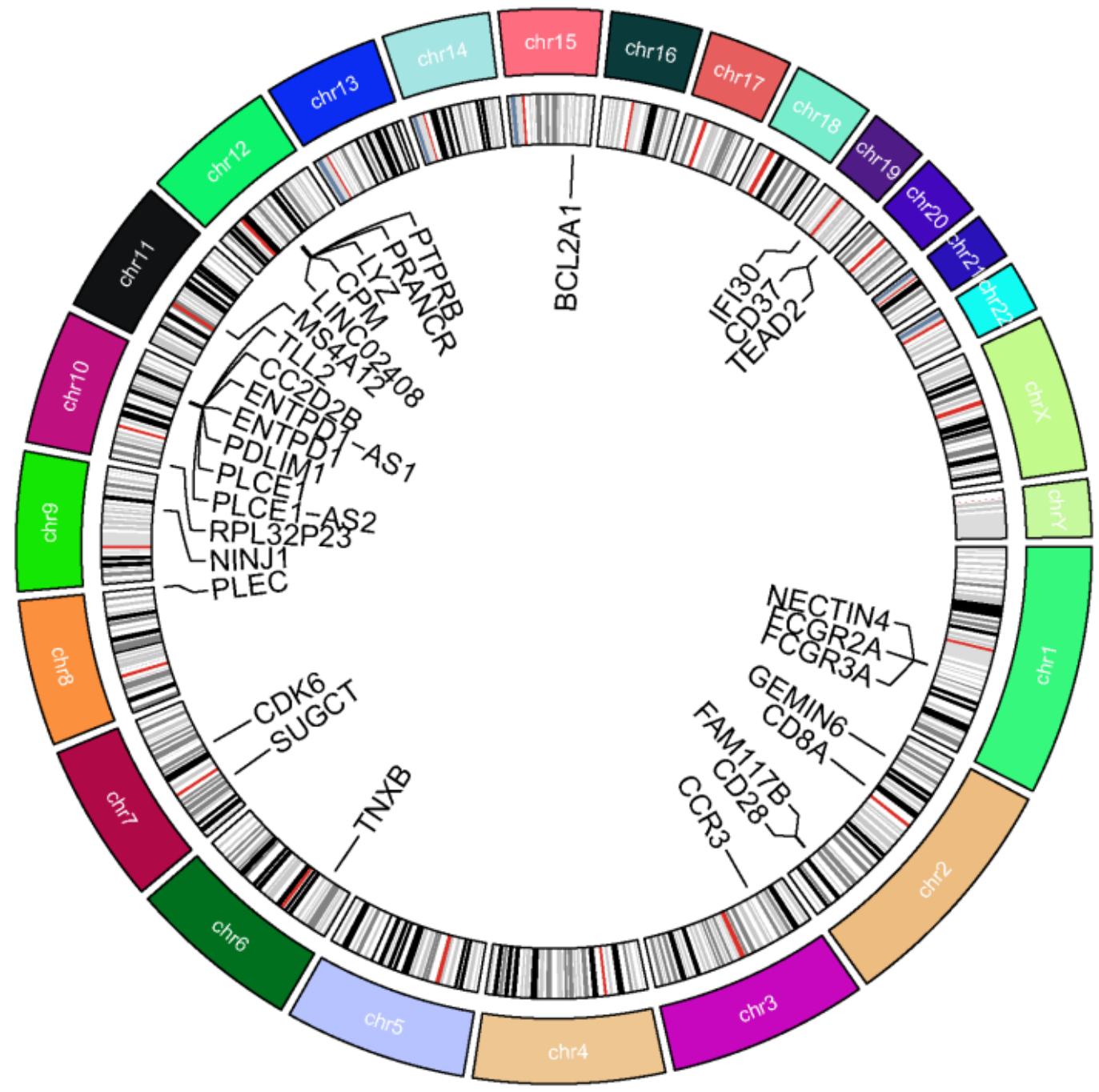
chr1
chr2
chr3
chr4
chr5
chr6
chr7
chr8
chr9
chr10
chr11
chr12
chr13
chr14
chr15
chr16
chr17
chr18
chr19
chr20
chrX
chrY
BCL2A1
PTPRB
PRANCR
LYZ
CPM
LINC02408
MS4A12
TLL2
CC2D2B
ENTPD1
ENTPD1-AS1
PDLIM1
PLCE1
PLCE1-AS2
RPL32P23
NINJ1
PLEC
IFI30
CD37
TEAD2
NECTIN4
FCGR2A
FCGR3A
GEMIN6
CD8A
FAM117B
CD28
CCR3
CDK6
SUGCT
TNXB

Fig. (S3)

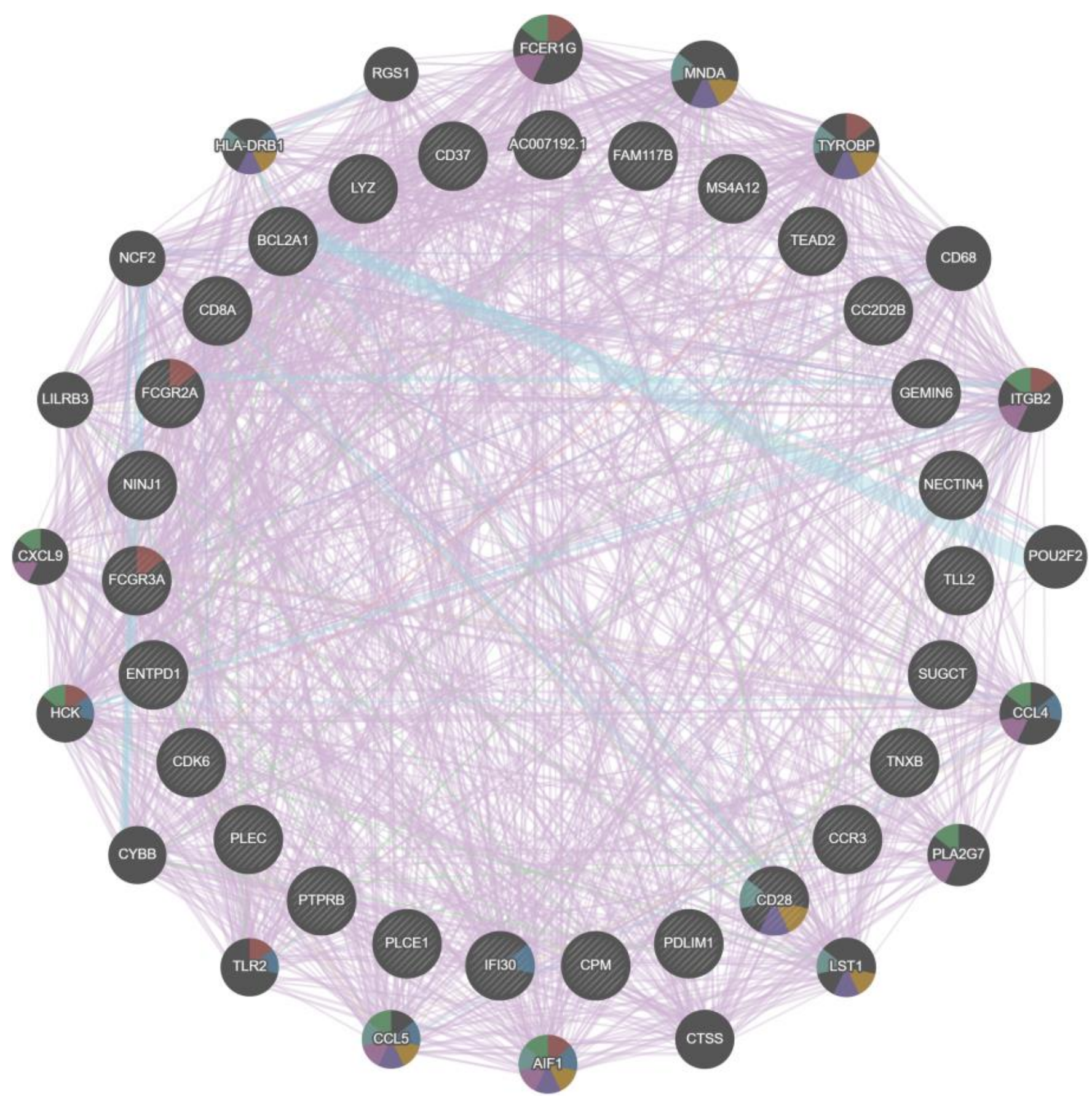
FCER1G
RGS1
MNDA
HLA-DRB1
CD37
AC007192.1
FAM117B
TYROBP
LYZ
MS4A12
BCL2A1
TEAD2
NCF2
CD68
CD8A
CC2D2B
LILRB3
FCGR2A
GEMIN6
ITGB2
NINJ1
NECTIN4
CXCL9
FCGR3A
POU2F2
TLL2
ENTPD1
SUGCT
HCK
CCL4
CDK6
TNXB
CYBB
PLEC
CCR3
PLA2G7
PTPRB
CD28
PLCE1
PDLIM1
TLR2
IFI30
CPM
LST1
CCL5
CTSS
AIF1

Fig. (S4)
